# The impact of floral morphology on genetic differentiation in two closely related biennial plant species

**DOI:** 10.1093/aobpla/ply051

**Published:** 2018-09-07

**Authors:** Arne Mertens, Rein Brys, Dorien Schouppe, Hans Jacquemyn

**Affiliations:** 1 Department of Biology, Plant Conservation and Population Biology, KU Leuven, Leuven, Belgium; 2 Research Institute for Nature and Forest, Geraardsbergen, Belgium

**Keywords:** AFLP, *Centaurium erythraea*, *Centaurium littorale*, floral morphology, geographic variation, herkogamy, isolation by distance, population structure

## Abstract

The genetic diversity and structure of plant populations are determined by the interaction of three distinct processes: gene flow, genetic drift and natural selection. These processes are to some extent dependent on the mating system of plants, which in turn is largely determined by floral morphology and the level of herkogamy in particular. In this study, we used molecular markers to investigate the impact of floral morphology on genetic differentiation and structure in two closely related *Centaurium* species that display large variation in floral morphology across two distinct geographic regions in Europe (mainland Europe and the UK). Our results showed that genetic differences between regions and populations within regions were similar for both species, but that patterns of genetic structure largely depended on the observed variation in floral morphology. Populations of *Centaurium erythraea* showed higher genetic differentiation and clear isolation by distance (IBD) in mainland Europe, but limited IBD in the UK. Opposite patterns were found in *Centaurium littorale*, with higher genetic differentiation and significant IBD in populations sampled in the UK and lower genetic differentiation in Continental populations with no pattern of IBD. Overall, these results indicate that variation in floral morphology has a profound impact on structuring of genetic diversity, with populations displaying low levels of herkogamy showing the strongest patterns of genetic structuring and the reverse pattern in populations showing high levels of herkogamy.

## Teaser

In this work, we compare the genetic differentiation and structure of populations of two closely related Centaurium species that display a large variation in floral morphology across two geographic regions in Europe. The populations of both species show opposite patterns of genetic differentiation and structure, and the patterns are reversed between the two regions. For both species, we find a strong link between these patterns and the differences in floral morphology observed in the sampled populations.

## Introduction

The genetic diversity and structure of plant populations are determined by the interaction of gene flow, genetic drift and natural selection, processes that are influenced by the geographic distribution of plant populations and population demography (Eckert *et al.* 2008). Historical events such as glaciation or orogeny determine to a large extent the geographic ranges of plant and animal species, which in turn are determined by geographic barriers that limit or prevent further dispersal (e.g. mountains, oceans, or more recently also fragmentation caused by human activities) (Slatkin 1987; Eckert *et al.* 2008; Zhang *et al.* 2014). Furthermore, environmental conditions often change gradually over a geographic gradient, not only determining the species that can live at a certain location, but also potentially leading to within-species variation by selecting for individuals that are genetically more adapted to specific conditions (Harrison 2006; Eckert *et al.* 2008). For example, temperature and humidity are strongly related to altitude or latitude and are both correlated with variation in plant functional traits, such as plant size (Li *et al.* 1998), leaf morphology (Meinzer *et al.* 1985; Byars *et al.* 2007) and flower morphology (Olsson and Ågren 2002; Levin 2010; 2012) within one species.

Differences in environmental pressures have been shown to influence plant mating systems, a major determinant of population genetic structure (Hamrick and Godt 1996; Arnaud-Haond *et al.* 2006; Honnay and Jacquemyn 2007; Aguilar *et al.* 2008; Soengas *et al.* 2013; Pettengill *et al.* 2016). Mating systems can show strong variation, even within a single species, ranging from fully outcrossing to entirely selfing (Schemske and Lande 1985; Barrett 2002; Goodwillie *et al.* 2005; Wright *et al.* 2013). Due to the effects of genetic drift, selfing populations are expected to be more homozygous and to be genetically less diverse than populations of outcrossing species (Schoen and brown 1991; Charlesworth and Charlesworth 1995; Williams 2001). Outcrossing populations, on the other hand, are expected to show reduced population structure due to higher gene flow via pollen (Stebbins 1957; Wright *et al.* 2013; Pettengill *et al.* 2016). As a result, outcrossing populations are more likely to show patterns of isolation by distance (IBD), with less gene flow between more distant populations. In contrast, a flat relationship with high variance between genetic and geographic distance can be expected when comparing selfing populations due to limited gene flow and the stochastic effects of genetic drift, even when populations are adjacent (Hutchison and Templeton 1999; Pettengill *et al.* 2016).

Selfing rates are strongly determined by flower characteristics such as flowering time and floral morphology (Wyatt 1982; Murawski and Hamrick 1992; Harder and Barrett 1996). One of the floral characteristics that has been demonstrated to have a major influence on selfing rates in self-compatible plants and to be under strong selection is herkogamy, i.e. the spatial separation of anthers and stigmas within a flower (Jacquemyn *et al.* 2012; Cheptou *et al.* 2017; Opedal *et al.* 2017; Toräng *et al.* 2017). Decreasing levels of stigma–anther segregation generally lead to higher rates of autonomous selfing (Karron *et al.* 1997; Brunet and Eckert 1998; Brys and Jacquemyn 2011; Brys *et al.* 2014). Geographic variation in the level of herkogamy can therefore be expected to have an impact on population genetic structure.

Changes in flower morphology and evolution to higher rates of selfing during range expansion as a mechanism for reproductive assurance might further reduce the genetic variation in plant populations (Wright *et al.* 2013; Griffin and Willi 2014). Particularly in populations that are located at the edges of a species’ range, it can be expected that their genetic structure deviates more strongly from that of core populations as populations at the edge of a species’ distribution often have lower population sizes and reduced genetic variation due to increased genetic drift and inbreeding, especially when migration from core populations is limited (Sagarin and Gaines 2002; Eckert *et al.* 2008; Pfeifer *et al.* 2009; Pouget *et al.* 2013). Additionally, repeated events of glaciation during the Quaternary period have resulted in the extinction of some and repeated colonization and range expansion from refugia of other species, shaping the genetic structure of populations and communities (Hewitt 2000; Voss *et al.* 2012; Cornille *et al.* 2013). Geographic range expansion during postglacial periods and the associated founding effects are expected to reduce allelic richness and increase homozygosity (Hewitt 2000; Pfeifer *et al.* 2009).

In this study, we investigated variation in genetic structure of two closely related *Centaurium* species that show large variation in floral morphology across two distinct regions in Europe (UK and mainland Europe) (Schouppe *et al.* 2017). In the UK, populations of *Centaurium erythraea* show lower levels of herkogamy while on the European mainland, populations show higher anther–stigma separation (Ubsdell 1979; Brys and Jacquemyn 2011; Schouppe *et al.* 2017). In contrast, *Centaurium littorale* shows smaller anther–stigma separation on the Continent (Brys and Jacquemyn 2011; Schouppe *et al.* 2017), while in the UK the overall level of herkogamy appears to be significantly higher (Ubsdell 1979; Schouppe *et al.* 2017). More specifically, we tested the hypothesis that populations showing low levels of herkogamy (*C. erythraea* in the UK, *C. littorale* in mainland Europe) display a stronger population genetic structure, but less pronounced patterns of IBD in comparison with more herkogamous populations (*C. littorale* in the UK, *C. littorale* in mainland Europe).

## Methods

### Study species

**Figure 1.**
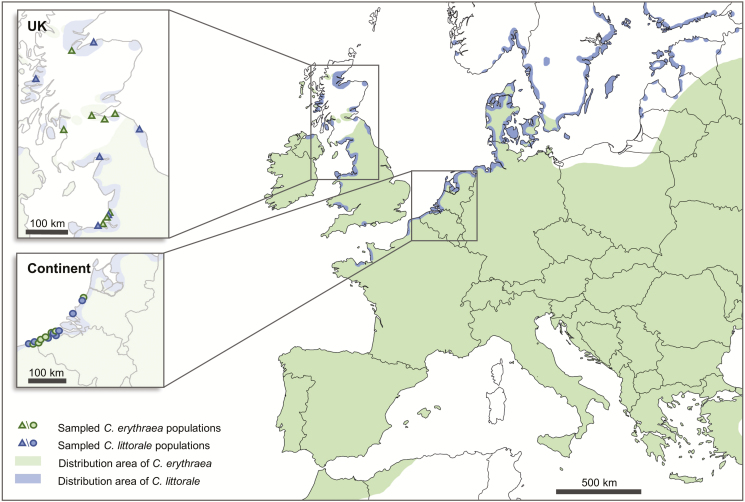
Distribution map of *Centaurium erythraea* (green) and *Centaurium littorale* (blue) including study area and sampled populations. Continental populations are denoted by circles while triangles represent populations in the UK.

*Centaurium erythraea* and *C. littorale* are both biennial, short-lived herbs that belong to the Gentianaceae. Although both species have a wide distribution in Europe and sometimes can be found in sympatry, they usually grow in different environments (Fig. 1). *Centaurium erythraea* shows by far the largest distribution area, ranging from the Mediterranean Basin in the south to Denmark and Scotland in the north. It can be found in habitats ranging from coastal dunes and river banks to wood margins and well-developed calcareous grasslands (Van Tooren *et al.* 1983; Schat *et al.* 1989). While *C. erythraea* prefers drier soils, *C. littorale* is more confined to coastal regions, with a higher tolerance to more humid soils, characterized by slightly saline conditions (Schat and Scholten 1985). The distribution of *C. littorale* ranges from the northwest of France to the coastline of the Scandinavian countries (Fig. 1).

Both species have pink showy and hermaphroditic flowers that are self-compatible. Flowering occurs from the end of June until the beginning of September (Ubsdell 1979; Brys *et al.* 2014), and individual flowers are only open for 4–5 days (Brys and Jacquemyn 2011). In mainland Europe, *C. erythraea* typically produces larger and more flowers than *C. littorale* (Brys and Jacquemyn 2011), whereas the opposite patterns were observed in populations of both species in the UK (Ubsdell 1979; Schouppe *et al.* 2017). Flowers of these *Centaurium* species do not produce any nectar and share the same set of floral visitors, mainly pollen gathering hoverflies (Diptera, Syrphidae), but also some bees (Hymenoptera, Apidae) and small flies (Empididae, Muscidae) (Brys and Jacquemyn 2011; Brys *et al.* 2011; Brys and Jacquemyn 2012). When suitable pollinators are absent, the breeding system of *C. erythraea* and *C. littorale* allows autonomous fruit and seed set by self-pollination through the curling of anthers at the end of anthesis (Brys and Jacquemyn 2011).

Flowers display variable levels of herkogamy, and significant species and region-dependent differences in the level of anther–stigma separation have been observed. High levels of herkogamy characterize flowers in Continental *C. erythraea* populations and low levels of herkogamy in UK populations, while opposite patterns have been described for *C. littorale* (Ubsdell 1979; Brys and Jacquemyn 2011; Schouppe *et al.* 2017).

### Study area and populations

To investigate geographical variation in population genetic structure in *C. littorale* and *C. erythraea*, 14 and 15 populations of each species were studied, respectively (Fig. 1). Fifteen populations were located in the UK (ranging from Scotland to Northern England and Wales) and 14 in mainland Europe (ranging from the North of the Netherlands to the North of France, including Belgium). A map with the population labels can be found in the supporting information **[see****Supporting Information—Fig. S1****]**. While *C. erythraea* has a larger ecological range than *C. littorale*, all populations were sampled in coastal areas and dune slacks with slightly saline and moist conditions to reduce the possibility that differences in genetic diversity and structure result from differences in biotic and abiotic conditions. This study consists of the same set of populations as sampled in Schouppe *et al.* (2017), but rather than comparing floral morphology, we add a molecular component to see whether differences in floral morphology are reflected in the way these populations are genetically structured.

### Amplified fragment length polymorphism fingerprinting

Leaf material was collected from 30 individuals of the 29 studied populations. Samples were taken from the entire area occupied by the population in order to avoid the effects of within-population substructure. Young leaf material was collected and immediately dried in silica gel. Before DNA extraction, leaf material was freeze-dried and homogenized with a mill (Retsch MM 200, Retsch GmbH, Haan, Germany) to a fine powder.

DNA was extracted using Isogen’s QuickPick DNA extraction kit (Isogen Life Science, De Meern, The Netherlands). Amplified fragment length polymorphism (AFLP) was used to genotype all individuals from each population using the standard protocol of Vos *et al.* (1995) with modifications by Brys *et al.* (2014). Four fluorescently labelled primer–enzyme combinations were used: E-ACC(NED)/M-CAA, E-ACC(VIC)/M-CTG, E-ACC(NED)/M-CAG and E-ACC(VIC)/M-CTC. They all had a scoring range of 150–600 bp and produced clear bands with enough variability for further analyses. Fragment length detection and separation of PCR products of the four primer–enzyme combinations were realized with an ABI 3500 capillary sequencer (Applied Biosystems™, Foster City, CA, USA) using Genescan 600-LIZ dye as a high density lane size standard (Applied Biosystems™, Foster City, CA, USA). Default parameters were used to size raw data with the GeneMapper 4.1 software (Applied Biosystems™, Foster City, CA, USA) and the data were scored with the RawGeno library of the R CRAN package (Arrigo *et al.* 2012) with a scoring range that covered 150–600 bp. Amplified fragment length polymorphism profiles were manually checked to discard profiles with large peak height variation and a filter parameter (cutRFU) of 400 was set to remove bins that displayed average fragment fluor intensity below this value. Mean error rates were estimated by adding differences between control profiles (a set of random samples that were genotyped twice) per primer–enzyme combination after bins with a frequency of <0.01 were eliminated.

The dominant (binary) data obtained from AFLP with the four different primer–enzyme combinations were further analysed. The number of polymorphic loci and expected heterozygosity for each population were determined using AFLP-SURV using a Bayesian method with non-uniform prior distribution, which is the default for non-haploid organisms to obtain the most accurate results (Zhivotovsky 1999). To incorporate the selfing capacity of plant species with a mixed-mating system, Wright’s inbreeding coefficient (*F*_is_) was set to 0.5 to account for an expected deviation from Hardy–Weinberg genotypic proportions (Vekemans 2002). Differences in the percentage of polymorphic loci and expected heterozygosity between populations and species in mainland Europe and the UK were investigated with a two-factor ANOVA, with species and geographic location as fixed effects.

### Large-scale population structure

Based on the AFLP data, population structure was estimated using an admixture model (i.e. allowing gene flow between clusters) in the software package STRUCTURE (Pritchard *et al.* 2000). Using a Bayesian framework, each individual was assigned to one or more clusters depending on the model used. In this analysis, an admixture model was run for 100000 iterations with a burn-in of 75000 iterations while using population IDs as sampling location information using the LOCPRIOR model. This was done for five iterations for a *K*-value (the number of allowed clusters) ranging from 1 to the total number of sampled populations (14 for *C. littorale* and 15 for *C. erythraea*). The optimal *K*-value was then determined by calculating Δ*K* as suggested by Evanno *et al.* (2005) with Structure Harvester (Earl and VonHoldt 2012), and was used to visualize an estimation of the population structure of both species in a bar plot. To assess whether large-scale population structure differed between species, two separate STRUCTURE analyses were run for all populations of each species.

To further investigate species-specific differences in genetic structure, an analysis of molecular variance (AMOVA) (Excoffier *et al.* 1992) was conducted. For both species, total genetic diversity was partitioned among regions (PhiRT), among populations within regions (PhiPR) and within populations in both regions (PhiPT) by carrying out a hierarchical AMOVA on Euclidean pairwise distances among individuals using GenAlEx. Pairwise mean population binary genetic distances and their significances were calculated using the Excel add-in tool GenAlEx v. 6.5 (Peakall and Smouse 2012). A principal coordinate analysis (PCoA) was performed to visualize the genetic distances between populations of both species.

### Isolation by distance

To assess the relative importance of genetic drift and gene flow between populations on genetic differentiation, the relationship between pairwise population genetic distance (φ_PT_) and the geographic distance separating these populations was evaluated with the Excel package GenAlEx (Peakall and Smouse 2012). φ_PT_ is an analogue of *F*_st_ that is often used when treating binary AFLP data. Pairwise population φ_PT_-value matrices were calculated with GenAlEx, using AMOVA with 9999 permutations implemented. Based on the geographical coordinates of all populations, log-transformed geographic distances between populations were calculated. Finally, a paired Mantel analysis was performed between the genetic distance matrices (φ_PT_) and the log-transformed geographic distance matrices for all populations of both species separately, split by region (UK or Continent).

### Linking herkogamy with genetic differentiation between populations

Herkogamy levels of each of the sampled populations were retrieved from Schouppe *et al.* (2017) and boxplots were created to compare the levels between species in each region. Similarly, pairwise genetic population distances (φ_PT_) were calculated using the AFLP data with the excel package ‘GenAlEx’ (Peakall and Smouse 2006) and values of among-population differentiation within each region were compared using a Student’s *t*-test after testing for normality and equal variances with Shapiro–Wilk and Levene’s tests, respectively.

## Results

### AFLP banding attributes

Four different primer–enzyme combinations resulted in a total number of 1935 scored loci of which 246 were polymorphic with allele frequencies varying between 1 and 99 % and mean error rates between 0.016 and 0.04 % **[see****Supporting Information—Table S1****]**. Expected heterozygosity levels ranged from 0.081 to 0.223 and number of polymorphic loci varied between 49 and 142 (Table 1). No significant differences in the number of polymorphic loci or expected heterozygosity levels were found between species (*P* = 0.894 and *P* = 0.502) or region (*P* = 0.549 and *P* = 0.347), respectively.

**Table 1.** Names, geographical coordinates and AFLP banding attributes of *Centaurium erythraea* and *Centaurium littorale* populations. *N*, number of scored individuals; Loc P, number of polymorphic loci at the 5 % level; PLP, proportion of polymorphic loci at the 5 % level; Hj, expected heterozygosity under Hardy–Weinberg genotypic proportions; SE (Hj), standard error of Hj.

Species	Name	Code	*n*	Loc P	PLP	Hj	SE (Hj)	Longitude	Latitude
European mainland									
*C. erythraea*	De Haan	DH	26	85	34.6	0.100	0.008	3°4′3″	51°17′33″
	Grenspad	GR	11	99	40.2	0.165	0.011	2°33′10″	51°4′48″
	Doornpanne	DP	23	87	35.4	0.122	0.010	2°39′13″	51°06′48″
	Groenplein	GP	10	72	29.3	0.130	0.010	3°19′8″	51°21′22″
	Meijendel	MD	23	69	28.0	0.096	0.009	4°19′27″	52°8′14″
	Ter Yde	TY	26	132	53.7	0.174	0.011	2°41′52″	51°8′10″
	Westhoek	WH	26	103	41.9	0.134	0.010	2°33′10″	51°5′15″
Average				92	37.6	0.132	0.010		
*C. littorale*	Brouwersdam	BD	21	50	20.3	0.084	0.008	3°51′25″	51°46′11″
	De Haan	DH	27	151	61.4	0.187	0.011	3°4′13″	51°17′45″
	Bray-Dunes	FR	12	136	55.3	0.194	0.011	2°31′52″	51°4′52″
	Zwin	CKN	24	87	35.4	0.105	0.009	3°20′9″	51°21′45″
	Groenplein	GP	4	137	55.7	0.140	0.011	3°19′8″	51°21′22″
	Meijendel	MD	28	92	37.4	0.110	0.009	4°19′27″	52°8′14″
	Ter Yde	TY	29	49	19.9	0.081	0.008	2°41′52″	51°8′10″
	Westhoek	WH	29	52	21.1	0.089	0.009	2°33′10″	51°5′15″
Average				94	38.3	0.124	0.010		
UK									
*C. erythraea*	Bo′ness	BN	22	124	50.4	0.154	0.010	−3°37′58″	56°0′58″
	Dirleton	DR	24	116	47.2	0.137	0.009	−2°48′25″	56°3′30″
	Formby	FB	16	96	39.0	0.158	0.011	−3°5′46″	53°34′0″
	Foyers	FO	29	83	33.7	0.092	0.008	−4°29′44″	57°15′22″
	Irvine	IR	21	75	30.5	0.096	0.008	−4°39′53″	55°35′53″
	Roslin	RS	24	81	32.9	0.112	0.010	−3°10′45″	55°51′32″
	Waterloo	WL	16	107	43.5	0.173	0.011	−3°2′20″	53°28′0″
	Ainsdale	AI	27	96	39.0	0.122	0.010	−3°3′10″	53°37′10″
Average				97	39.5	0.131	0.010		
*C. littorale*	Holy Island	HI	26	97	39.4	0.127	0.009	−1°50′3″	55°41′5″
	Kentra	KT	19	99	40.2	0.130	0.009	−5°51′3″	56°45′27″
	Kinloss	UKN	24	152	61.8	0.223	0.012	−3°35′18″	57°38′56″
	Powfoot	PF	23	121	49.2	0.160	0.010	−3°22′0″	54°58′17″
	Ainsdale	AI	24	116	47.2	0.126	0.009	−3°3′10″	53°37′10″
	Talacre	TA	28	76	30.9	0.087	0.008	−3°19′57″	53°21′16″
Average				110	44.8	0.142	0.010		

### Population genetic structure

**Figure 2.**
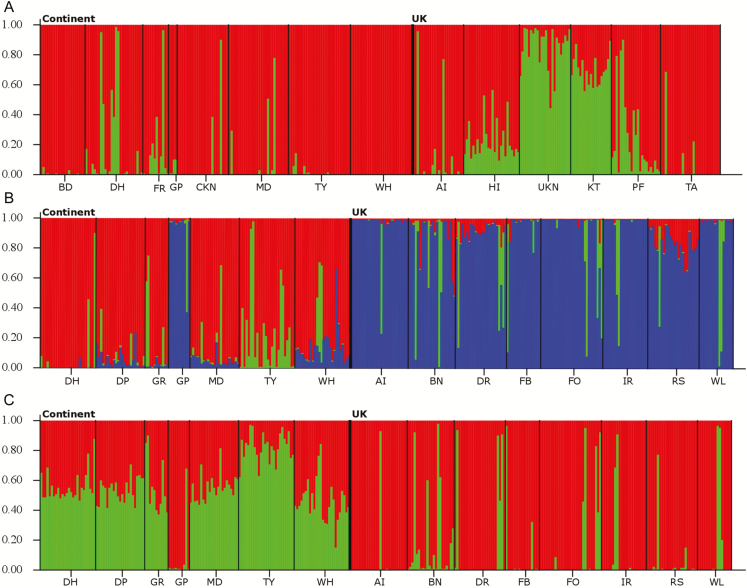
Structure analyses on populations of two *Centaurium* species. (A) *Centaurium littorale* at *K* = 2; (B) and (C) *Centaurium erythraea* at *K* = 2 and *K* = 3, respectively. The thick line represents the separation of populations sampled in mainland Europe and in the UK.

Structure Harvester found highest Δ*K* values for *K* = 2 for *C. littorale* and *K* = 3 for *C. erythraea***[see****Supporting Information—Table S2****]**. Differences in the way populations were clustered could be distinguished between the two geographic regions. In *C. littorale* sampled in mainland Europe (Fig. 2A), most individuals of each population were assigned to one single cluster. The opposite pattern was observed for *C. littorale* populations sampled in the UK, where almost no individual belonged to a single cluster, with all individuals of populations HI, UKN, KT and PF assigned to both clusters. Populations sampled at AI and TA, however, appeared to cluster with the mainland populations. Similarly, clear differences in clustering patterns for *C. erythraea* populations could be distinguished between the two regions for *K* = 3, with individuals of populations in the UK almost entirely assigned to a different cluster than individuals of Continental populations (Fig. 2B). When the number of clusters was set to *K* = 2, almost all individuals of Continental populations were assigned to both clusters, while most individuals of populations in the UK were assigned to just one cluster (Fig. 2C), suggesting that only a set of the genetic diversity present in mainland Europe was present in the UK. The mainland population sampled at GP, however, almost entirely grouped with the UK populations.

**Figure 3.**
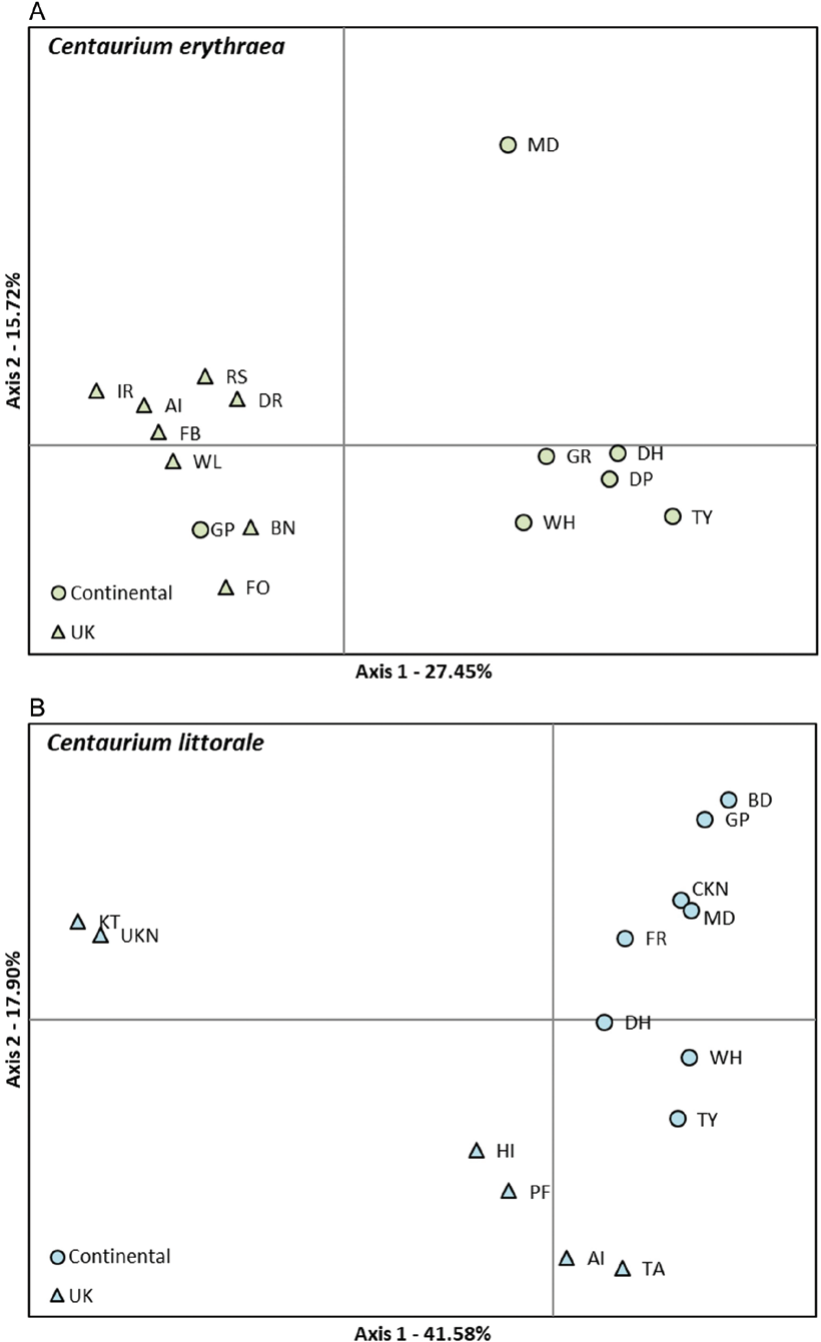
Principal coordinate analysis based on pairwise mean population binary genetic distances of sampled *Centaurium erythraea* (A) and *Centaurium littorale* (B) populations. Triangles represent populations in the UK and circles represent populations in mainland Europe.

The PCoA showed that the two species displayed clear genetic differentiation along the first axis, which explained 27.45 % of the observed variation in *C. erythraea* (Fig. 3A) and 41.58 % in *C. littorale* (Fig. 3B) and separated to a large extent Continental and UK populations of *C. erythraea*. The second axis explained 15.72 and 17.90 % of the observed variation for *C. erythraea* and *C. littorale*, respectively, and separated to some extent the population at MD from other Continental populations of *C. erythraea*. One population of *C. erythraea* (GP) clustered with UK populations rather than with the other Continental populations (Fig. 3A). The two *C. littorale* populations that were sampled in Scotland (UKN, KT) were genetically more distant to the other *C. littorale* populations (Fig. 3B).

Pairwise population genetic distances varied considerably, between 0.082 and 0.564 for *C. erythraea* and between 0.027 and 0.561 for *C. littorale***[see****Supporting Information—Tables S3****and****S4****]**. Populations of *C. erythraea* were significantly more differentiated (*P* = 0.048) from each other in mainland Europe than in the UK (average 0.311 and 0.250, respectively) **[see****Supporting Information—Table S3****]**. The average among-population genetic distance between Continental *C. littorale* populations was 0.198 and 11 out of 28 comparisons had a φ_PT_ < 0.15 **[see****Supporting Information—Table S4****]**. In the UK, on the other hand, *C. littorale* populations were significantly more differentiated from each other with an average pairwise genetic distance of 0.304, although two populations (UKN, KT) were remarkably similar to each other (φ_PT_ = 0.073). For both species, among-population variation was highest when comparing populations of both regions (average φ_PT_ = 0.348 for *C. erythraea* and φ_PT_ = 0.333).

The AMOVA indicated that *C. littorale* and *C. erythraea* differed significantly between both regions, among populations within regions and within populations among all populations (Table 2). For both species, the highest percentage of genetic variation was found within populations, followed by variation among populations within regions and among regions, respectively.

**Table 2.** PhiRT (among-region variation), PhiPR (among-population variation within region) and PhiPT (total variation within all populations) and *P*-values for sampled populations of *Centaurium erythraea* and *Centaurium littorale* including the percentage of variation that is explained by each.

	*C. erythraea*			*C. littorale*		
	Value	*P*(rand ≥ data)	% of var	Value	*P*(rand ≥ data)	% of var
PhiRT	0.100	0.0001	10 %	0.102	0.0001	10 %
PhiPR	0.286	0.0001	26 %	0.256	0.0001	23 %
PhiPT	0.358	0.0001	64 %	0.332	0.0001	67 %

### Isolation by distance

**Figure 4.**
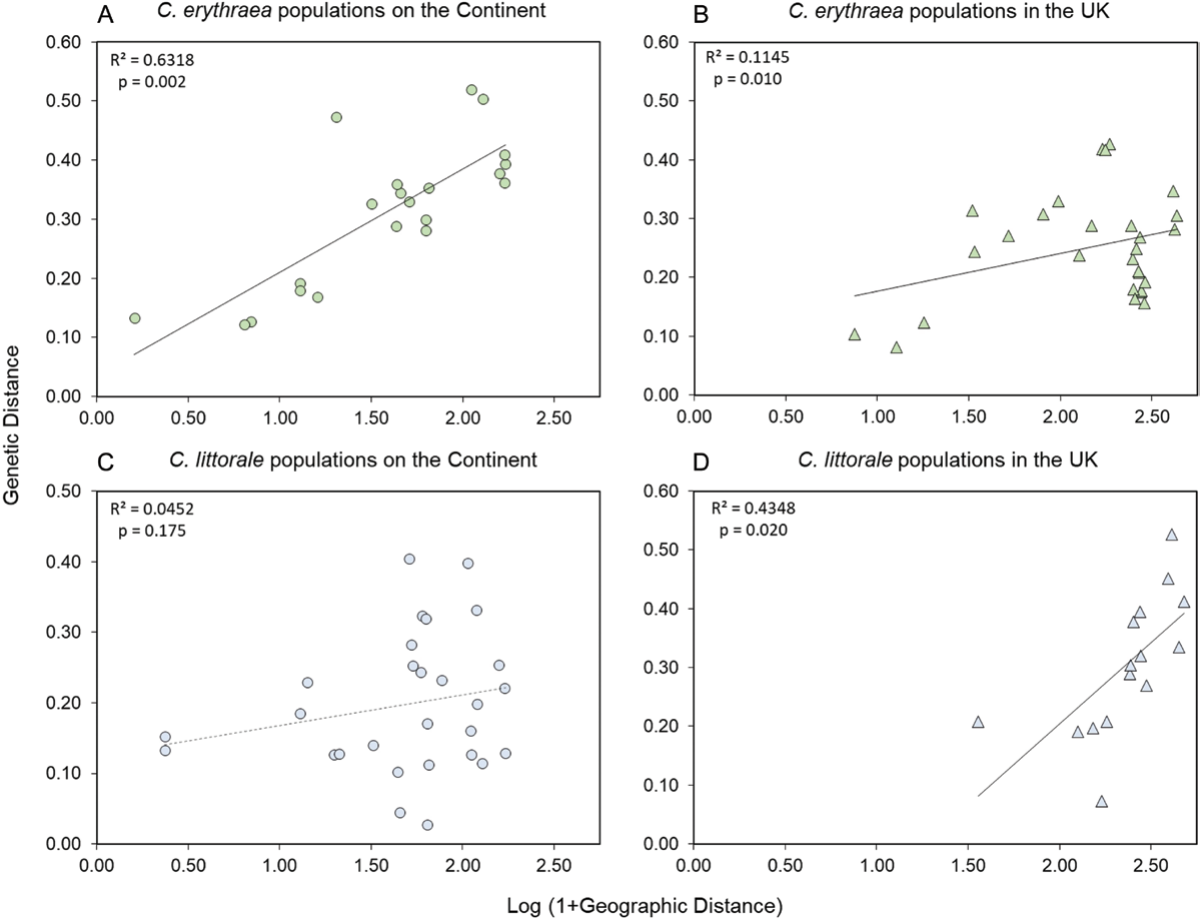
Correlation between pairwise population φ_PT_ values and geographic distances between populations. (A and B) Continental and UK populations of *Centaurium erythraea*, respectively. (C and D) Continental and UK populations of *Centaurium littorale*, respectively. Non-significant correlations are represented by a dashed line.

Correlations between geographic distance and pairwise genetic distances (φ_PT_) were significant for *C. erythraea* populations both in mainland Europe and in the UK (Fig. 4A and B), but the relationship was much stronger in mainland Europe (with 63.2 % of the variation explained by the regression curve) compared to the UK (with only 11.5 % of the variation explained). No significant correlation between geographic and genetic distance was found in Continental populations of *C. littorale* (Fig. 4C), whereas in the UK, this correlation was highly significant, with 43.5 % of the variation explained by the regression line (Fig. 4D).

### Herkogamy and pairwise genetic population distances

**Figure 5.**
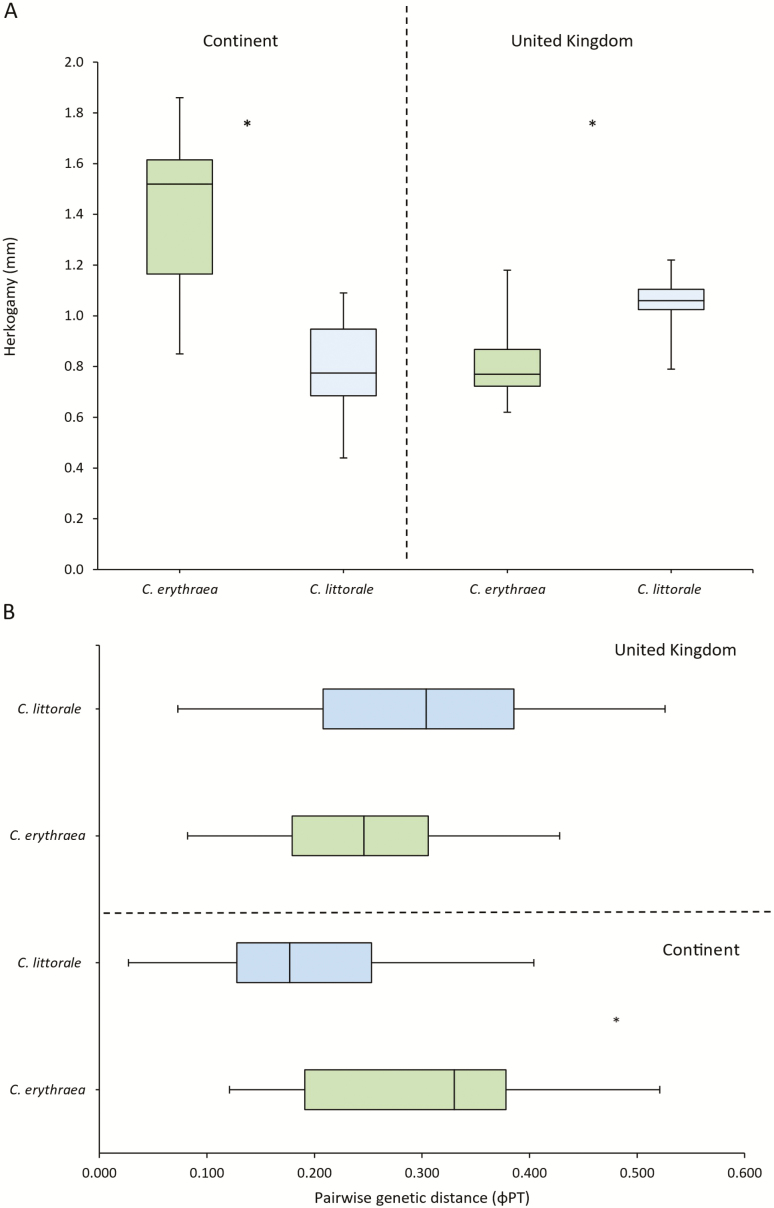
Boxplots comparing levels of herkogamy (A) and levels of genetic differentiation (B) between populations of both species in two distinct areas. Significant differences are marked with an asterisk.

In general, populations of *C. erythraea* in the European mainland showed significantly (*p* < 0.001) higher levels of herkogamy than *C. littorale* populations (1.38 and 0.79 mm, respectively). In contrast, in UK populations of *C. littorale*, populations had significantly higher levels of herkogamy (*P* = 0.01) than populations of *C. erythraea* (1.047 and 0.815 mm, respectively) (Fig. 5A). Similarly, pairwise genetic distances between of *C. erythraea* populations in the European mainland were higher (*P* < 0.001) than those between *C. littorale* populations (φ_PT_ = 0.311 and φ_PT_ = 0.198, respectively). Genetic distances between UK populations of *C. erythraea* are lower than those between *C. littorale* populations (φ_PT_ = 0.250 and φ_PT_ = 0.304, respectively), but the difference was not significant (*P* = 0.104) (Fig. 5B).

## Discussion

It is generally expected that the geographic distribution and mating system of plant species can shape their population genetic diversity and structure via processes such as gene flow, genetic drift and natural selection (Loveless and Hamrick 1984; Hamrick and Godt 1996; Honnay and Jacquemyn 2007; Aguilar *et al.* 2008; Eckert *et al.* 2008; Soengas *et al.* 2013; Pettengill *et al.* 2016). In this study, we conducted an extensive population genetic study on the same set of populations of two closely related *Centaurium* species that were sampled in Schouppe *et al.* (2017) and that showed strong geographic variation in floral morphology (Ubsdell 1979; Brys and Jacquemyn 2011; Schouppe *et al.* 2017).

Clear differences in population genetic structure were found between UK and Continental populations in both *Centaurium* species. While Structure was not able to clearly distinguish the sampled populations when the number of clusters was set equal to the number of studied populations, it distinguished UK and Continental populations when the number of clusters was set to *K* = 2 and *K* = 3 for *C. littorale* and *C. erythraea*, respectively, as suggested by Structure Harvester. More interestingly, the observed patterns of genetic structuring in both regions were reversed for both species. Differences in the level of herkogamy between the two species and regions mirror to a large extent the observed patterns of genetic structure, with high levels of genetic structuring in populations with lower herkogamy levels and low genetic structuring in more herkogamous populations. Pettengill *et al.* (2016) similarly found strong population structure in selfing populations of *Clarkia xantiana*, without patterns of IBD in comparison with more outcrossing populations that were less genetically structured but showed a higher IBD relationship.

Two populations (AI and TA) of *C. littorale*, however, clustered rather within the populations sampled on the European mainland. The fact that both species co-occur in these two populations might partially explain altered patterns of genetic structure. Because both species share the same set of pollinators, living in sympatry might induce a shift floral traits, with subsequent changes in the degree of selfing as result to assure reproduction. Such shifts in the level of selfing have been observed in multiple plant species, including *Centaurium* (Grossenbacher and Whittall 2011; Briscoe Runquist and Moeller 2014; Schouppe *et al.* 2017). Dominant data however make it hard to validate whether observed patterns are really caused by increased levels of selfing, because inbreeding coefficients cannot be calculated properly. Both in the PCoA and structure analyses, the population of *C. erythraea* sampled at GP clustered rather with UK populations than with other mainland populations. This population is however not geographically more isolated from the other populations and it is unlikely to assume that a dispersal event from the UK to the mainland occurred to found this population.

Clear patterns of IBD in Continental populations of *C. erythraea* indicate that adjacent populations are genetically more similar to each other and that dispersal limitation rather than genetic drift is the main determinant of genetic structure (Hutchison and Templeton 1999). Although *C. erythraea* populations in the UK also showed significant patterns of IBD, the relationship was much weaker (less than five times of the observed variation that could be explained by the regression curve), while the sampled populations in the UK were more geographically distant to each other. Reduced levels of herkogamy were found in the same UK populations (Schouppe *et al.* 2017) and subsequent increased selfing can be expected to lead to less gene flow among nearby populations, increasing population differentiation and less stringent relationships between genetic and geographic distances (Hamrick and Godt 1990; Pettengill *et al.* 2016). In *C. littorale*, on the other hand, Continental populations did not show any IBD effect, suggesting either high or very limited gene flow between these populations (Hutchison and Templeton 1999). Given the relatively large scatter of pairwise genetic distances and the smaller distances separating populations, this can probably be explained by the higher selfing behaviour of these populations, caused by the lack of herkogamy (Karron *et al.* 1997; Brunet and Eckert 1998; Brys and Jacquemyn 2011).

The observed differences in floral morphology and genetic structure can to some extent be related to the geographic range of both species. *Centaurium erythraea* shows a much larger distribution area, which encompasses a large part of the Mediterranean and Continental Europe in comparison with the more restricted distribution of *C. littorale. Centaurium erythraea* populations sampled in this study were located in the same habitat as *C. littorale* but they are not strictly confined to a coastal distribution and generally prefer drier soils (Schat and Scholten 1985). In a phylogeographic context of postglacial colonization, *C. erythraea* might have evolved to a more selfing mating system in the UK, especially in the suboptimal habitat near the coastlines to assure reproduction. This assumption is supported by the fact that for both species, geographic variation has been described in other characteristics that are expected to change during the evolution of outcrossing to selfing, such as nectar production, selfing capacity and flower morphology (Ornduff 1969). Because *C. littorale* was already confined to a coastal distribution in mainland Europe, recolonization of the UK might not have induced a similar trend in this species compared to *C. erythraea.*

## Conclusions

Our analyses of genetic structure revealed two interesting patterns. Clear within-species differences in genetic structure were found between UK populations and populations in mainland Europe and this pattern was reversed between the two species. We propose that observed differences in mating system contributed to a large extent to the observed variation in genetic structure, with the lowest differentiation and highest genetic structure in populations that showed the lowest herkogamy levels. Similarly, significant patterns of IBD were found in populations with higher anther–stigma separation while this correlation was reduced in less herkogamous populations. However, more extensive studies on mating system functioning, pollen transfer and dispersal patterns are needed to investigate in more detail the effect of selfing and patterns of gene flow within and between populations on the spatial genetic structure in both species. Additional research with co-dominant markers is necessary to give more detailed information on population-specific levels of inbreeding and their relationship with variation in floral morphology.

## Sources of Funding

This work was supported by the Research Foundation – Flanders (FWO) (G.0982.13).

## Contributions by the Authors

H.J. and R.B. designed the study, D.S. collected the samples, H.J. and A.M. analysed the data, A.M. wrote the first draft of the paper, all authors read and approved the final draft.

## Conflict of Interest

None declared.

## Supporting Information

The following additional information is available in the online version of this article—

**Figure S1.** Labelled distribution map of *Centaurium erythraea* (green) and *Centaurium littorale* (blue) including study area and sampled populations. Continental populations are denoted by circles while triangles represent populations in the UK.

**Table S1.** Used primer–enzyme combinations for amplified fragment length polymorphism (AFLP) barcoding and the initial number of loci, number of polymorphic loci and mean error rate for each of these combinations.

**Table S2**. Determining the optimal *K*-value for *Centaurium erythraea* and *Centaurium littorale* populations by calculating Δ*K* with Structure Harvester.

**Table S3.** Pairwise φ_PT_ values between *Centaurium erythraea* populations.

**Table S4**. Pairwise φ_PT_ values between *Centaurium littorale* populations.

Supporting Material
