# Supplementary material for: The impact of floral morphology on genetic differentiation in two closely related biennial plant species
Source: AoB Plants. 2018 Sep 7;10(5):ply051. doi: 10.1093/aobpla/ply051 (PMC6178171; doi:10.1093/aobpla/ply051)
Supplement: Supporting Material [file ply051_suppl_supporting_material.pdf]

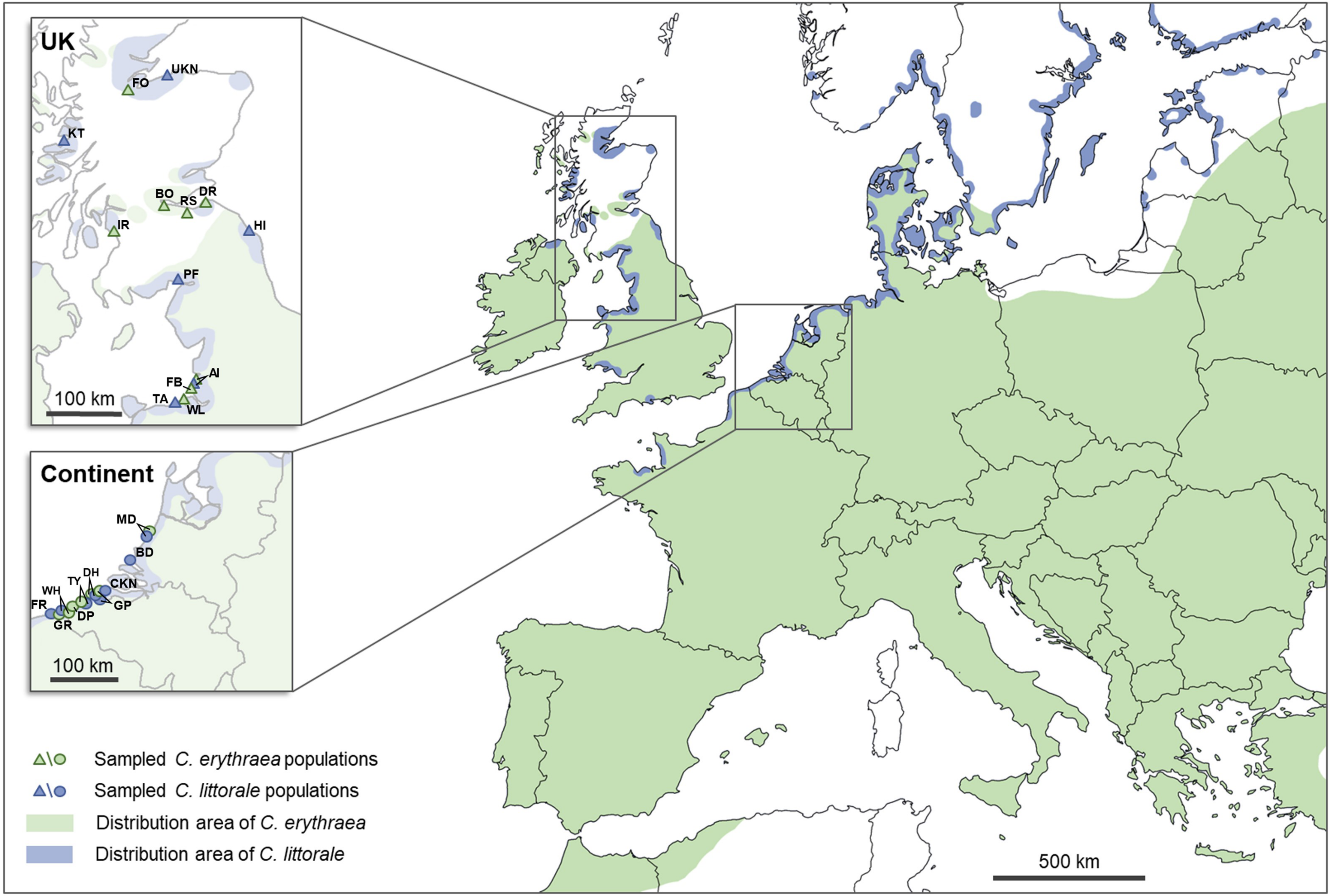

Figure S1. Labelled distribution map of *C. erythraea* (green) and *C. littorale* (blue) including study area and sampled populations. Continental populations are denoted by circles while triangles represent populations in the UK.

Table S1: Used primer-enzyme combinations for AFLP barcoding and the initial number of loci, number of polymorphic loci and mean error rate for each of these combinations.

	**Primer-enzyme combination**	**Initial number of loci**	**Number of polymorphic loci with allele frequencies between 1-99%**	**Mean error rate (%)**
**PC1**	E-ACC(NED)/M-CAA	493	62	0.0161
**PC2**	E-ACC(VIC)/M-CTG	539	78	0.0385
**PC3**	E-ACC(NED)/M-CAG	490	57	0.0351
**PC4**	E-ACC(VIC)/M-CTC	413	49	0.04
**Total**		**1935**	**246**	

Table S2: Determining the optimal *K*-value for *C. erythraea* and *C. littorale* populations by calculating Delta *K* with Structure Harvester.

Centaurium erythraea

K	Reps	Mean LnP(K)	Stdev LnP(K)	Ln'(K)	|Ln''(K)|	Delta K
**1**	5	-19610	0.254951	—	—	—
**2**	5	-17499.3	10.81883	2110.72	679.34	62.79239
**3**	5	-16067.9	4.828561	1431.38	735.38	152.298
**4**	5	-15371.9	63.08094	696	15.62	0.247618
**5**	5	-14691.5	57.39301	680.38	85.44	1.488683
**6**	5	-14096.6	128.839	594.94	135.5	1.0517
**7**	5	-13637.1	156.8605	459.44	134.16	0.855282
**8**	5	-13311.9	195.3006	325.28	36.48	0.186789
**9**	5	-12950.1	127.028	361.76	154.66	1.217527
**10**	5	-12743	213.1576	207.1	74.52	0.3496
**11**	5	-12461.4	65.92649	281.62	69.46	1.053598
**12**	5	-12249.2	96.06184	212.16	477.4	4.969715
**13**	5	-12514.5	981.3655	-265.24	870.42	0.886948
**14**	5	-11909.3	83.53105	605.18	593.68	7.107297
**15**	5	-11897.8	381.8612	11.5	1042.72	2.730626
**16**	5	-12929	1829.757	1031.22	—	—

Centaurium littorale

K	Reps	Mean LnP(K)	Stdev LnP(K)	Ln'(K)	|Ln''(K)|	Delta K
**1**	5	-19395.8	0.286356	—	—	—
**2**	5	-16311.7	2.917705	3084.12	2165.34	742.1382
**3**	5	-15392.9	37.73343	918.78	55.62	1.474024
**4**	5	-14529.7	608.8231	863.16	98.04	0.161032
**5**	5	-13568.5	47.10241	961.2	438.06	9.300161
**6**	5	-13045.4	1.710848	523.14	305.5	178.5664
**7**	5	-12827.7	27.47896	217.64	28.06	1.021145

**8**	5	-12638.2	17.92339	189.58	17.76	0.990884
**9**	5	-12466.3	61.76154	171.82	6.5	0.105243
**10**	5	-12301	65.10136	165.32	133.98	2.058022
**11**	5	-12269.7	148.4126	31.34	437.02	2.944628
**12**	5	-12675.4	1046.411	-405.68	977.28	0.933935
**13**	5	-12103.8	94.44249	571.6	1481.08	15.68235
**14**	5	-13013.2	1870.861	-909.48	719.06	0.384347
**15**	5	-13203.7	1844.354	-190.42	—	—

Table S3: Pairwise φ_{PT} values between *Centaurium erythraea* populations

	DH	DP	GR	GP	MD	TY	WH	AI	BN	DR	FB	FO	IR	RS	WL
**DH**	0.000														
**DP**	0.344	0.000													
**GR**	0.359	0.126	0.000												
**GP**	0.474	0.352	0.280	0.000											
**MD**	0.504	0.394	0.362	0.521	0.000										
**TY**	0.326	0.168	0.191	0.330	0.378	0.000									
**WH**	0.287	0.121	0.132	0.299	0.411	0.179	0.000								
**AI**	0.454	0.333	0.298	0.275	0.408	0.347	0.276	0.000							
**BN**	0.408	0.292	0.218	0.236	0.437	0.267	0.226	0.207	0.000						
**DR**	0.392	0.328	0.287	0.264	0.417	0.339	0.235	0.268	0.271	0.000					
**FB**	0.384	0.282	0.250	0.233	0.399	0.306	0.232	0.103	0.175	0.177	0.000				
**FO**	0.533	0.406	0.414	0.394	0.564	0.374	0.334	0.346	0.288	0.419	0.282	0.000			
**IR**	0.521	0.440	0.399	0.354	0.501	0.419	0.360	0.288	0.308	0.237	0.231	0.428	0.000		
**RS**	0.466	0.319	0.325	0.356	0.430	0.336	0.329	0.180	0.243	0.314	0.164	0.418	0.330	0.000	
**WL**	0.384	0.268	0.235	0.212	0.411	0.299	0.225	0.123	0.156	0.192	0.082	0.305	0.249	0.210	0.000

Table S4: Pairwise φ_{PT} values between *Centaurium littorale* populations.

	BD	DH	FR	GP	CKN	MD	TY	WH	AI	HI	UKN	KT	PF	TA
**BD**	0.000													
**DH**	0.232	0.000												
**FR**	0.198	0.045	0.000											
**GP**	0.323	0.126	0.027	0.000										
**CKN**	0.243	0.128	0.111	0.152	0.000									
**MD**	0.253	0.114	0.128	0.127	0.160	0.000								
**TY**	0.398	0.140	0.228	0.404	0.282	0.253	0.000							
**WH**	0.331	0.102	0.132	0.319	0.170	0.221	0.184	0.000						
**AI**	0.416	0.167	0.211	0.335	0.294	0.306	0.289	0.258	0.000					
**HI**	0.388	0.207	0.222	0.321	0.333	0.315	0.340	0.343	0.290	0.000				
**UKN**	0.438	0.263	0.252	0.298	0.372	0.378	0.426	0.409	0.334	0.304	0.000			
**KT**	0.561	0.359	0.377	0.501	0.482	0.493	0.546	0.516	0.451	0.394	0.073	0.000		
**PF**	0.360	0.169	0.152	0.240	0.274	0.249	0.296	0.271	0.197	0.191	0.269	0.377	0.000	
**TA**	0.464	0.221	0.256	0.412	0.314	0.321	0.300	0.270	0.208	0.320	0.412	0.526	0.208	0.000
